# Interim FDG-PET/CT for therapy monitoring and prognostication in Hodgkin’s Lymphoma

**DOI:** 10.1038/s41598-022-22032-3

**Published:** 2022-10-21

**Authors:** Akram Al-Ibraheem, Farah Anwer, Malik E. Juweid, Qaid Ahmed Shagera, Aysar N. Khalaf, Shahed Obeidat, Areen Mansour, Mohammad Ma’koseh, Khalid Halahleh, Imad Jaradat, Nidal Almasri, Asem Mansour

**Affiliations:** 1 Department of Nuclear Medicine and PET/CT, King Hussein Cancer Center, P.O. Box 1269, Al- Jubeiha, Amman, 11941 Jordan; 2 School of Medicine, the University of Jordan, Amman, Jordan; 3 Department of Nuclear Medicine, Warith International Cancer Institute, Karbala, Iraq; 4 Department of Nuclear Medicine, Institut Jules Bordet, Universite Libre de Bruxelles (ULB), Bruxelles, Belgium; 5 Department of Medical Oncology, King Hussein Cancer Center, Amman, Jordan; 6 Department of Radiation Oncology, King Hussein Cancer Center, Amman, Jordan; 7 Department of Pathology, King Hussein Cancer Center, Amman, Jordan; 8 Department of Diagnostic Radiology, King Hussein Cancer Center, Amman, Jordan

**Keywords:** Cancer, Diseases, Medical research, Oncology

## Abstract

The aim of the study was to assess the predictive value of interim FDG-PET/CT (iPET) in patients with Hodgkin’s lymphoma (HL) treated with Adriamycin, bleomycin, vinblastine and dacarbazine (ABVD) chemotherapy. A total of 245 consecutive patients with de novo HL between 12/2013 and 12/2017 were evaluated retrospectively. All patients were treated with upfront ABVD, performed PET/CT scans at baseline, after 2 cycles (interim PET, iPET2) or 4 cycles (iPET4) and at the end of therapy, and followed up for at least 6 months after therapy. The response status on iPET was defined according to the standard five-point Deauville scores (DS) as follows: complete metabolic response (CMR, DS 1–3) and non-complete metabolic response (nCMR) (DS 4 and 5). End-of-treatment (EoT) response was assessed by FDG-PET/CT and if needed biopsy confirmation of PET-positive findings. The association between iPET and EoT response was investigated using logistic regression analysis. Survival analysis was performed using the Cox regression hazard model and Kaplan–Meier methods. Sixty-nine patients underwent iPET-2 and 176 iPET-4. No association was found between the timing of iPET and iPET response status (*P*-value = 0.71). Two hundred and one patients (82%) had iPET-CMR and 44 (18%) iPET -nCMR. iPET was strongly associated with EoT response status: 194/201 (96 .5%) of iPET-CMR had a complete response at the EoT while only 21/44 (47.7%) of patients with iPET-nCMR presented a complete response at EoT (*P*-value < 0.0001). The median follow-up was 32 months (range 6–81). Patients with iPET-CMR presented a better outcome with 91% 3 y event-free-survival (EFS) and 95% 3 y overall survival (OS) than those with iPET-nCMR (41 and 86%, respectively, *P*-value < 0.0001). In multivariable analyses, iPET retained an independent prognostic factor of EFS and OS (*P*-value < 0.0001 and *P*-value = 0.002, respectively). iPET is highly predictive of outcome of HL patients treated with ABVD and allows to tailor therapy to the individual patient.

## Introduction

Fluorine-18-fluorodeoxyglucose (FDG) positron emission tomography/computed tomography (PET/CT) is routinely recommended for initial staging, re-staging and recurrence detection of Hodgkin’s lymphoma (HL) with an impact on patient management^1–3^. Interim FDG-PET/CT (from thereon referred to as iPET) is typically performed after 2–4 cycles of chemotherapy is now also commonly performed in patients with HL based on several studies demonstrating its prognostic value following standard therapy with the potential for treatment escalation or de-escalation based on the scan results^1,2,4–9^.

Most studies on the prognostic value of iPET in HL performed without alteration of treatment based on iPET were conducted prior to standardization of scan interpretation using the now widely Deauville criteria^5–9^. On the other hand, the vast majority of reported studies employing the Deauville criteria in iPET in HL used these criteria to alter treatment based on the iPET scan making it very difficult to determine the true predictive value of iPET without the scan-based intervention^10–15^. Thus, there is a need to fully explore the predictive value of iPET in HL using the contemporary Deauville criteria without changing treatment based on the scan results, particularly with respect to its reportedly variable positive predictive value (PPV)^16^.

In the current study, we assessed the predictive value of iPET interpreted using the Deauville criteria in a relatively large number of HL patients treated with Adriamycin, bleomycin, vinblastine and dacarbazine (ABVD) chemotherapy, the most widely used regimen in both early and advanced HL with no treatment change made based on iPET. In particular, we report on the correlation between the iPET findings after 2 or 4 cycles of ABVD and end-of-therapy (EoT) response, event-free and overall survival (EFS and OS). It is important to stress that our HL population is homogenous with respect to ethnicity; the vast majority of those patients are Jordanian Arabs, a population that has not been well-studied before.

## Materials and methods

### Patients

This was a single-centre retrospective study conducted at the King Hussein Cancer Center (KHCC) in Amman, Jordan and approved by the Institutional Review Board (IRB) of KHCC (IRB: 19 KHCC 37).

The inclusion criteria were as follows: (a) diagnosed with de novo classic HL (cHL), (b) treated with upfront ABVD given for 4–8 cycles, (c) underwent three times FDG-PET/CT at baseline, post cycles 2 or 4 of ABVD (iPET) and at the end of therapy (EoT-PET) and (d) followed-up for at least 6 months after chemotherapy. Exclusion criteria were patients with any history of prior lymphoma and the presence of second cancer (aside from basal cell carcinoma). The known risk factors in HL including clinical, demographic and laboratory factors were retrieved from the patient medical records. Laboratory factors were categorized according to the thresholds in the NCCN guideline (serum albumin level of less than 4 g per deciliter, a hemoglobin level of less than 10.5 g per deciliter, leukocytosis (a white-cell count of at least 15,000 per cubic millimeter), and lymphocytopenia (a lymphocyte count of less than 600 per cubic millimeter, a count that was less than 8 percent of the white-cell count, or both)^17^. Disease staging was performed using FDG-PET/CT, conventional imaging, clinical examination and bone marrow biopsy. Bone marrow biopsy was repeated at the end of the treatment if there was an initial bone marrow involvement.

The disease stage was assigned based on the Anne-Arbor staging system. Bulky mass was defined according to NCCN guidelines as the presence of a mass ≥ 10 cm in diameter^17^.

Patients with early-stage HL were given 4–6 cycles of ABVD. Patients with the advanced-stage disease were treated with 6 to 8 cycles of ABVD. Radiotherapy was given to bulky disease sites with megavoltage energies to tumour doses of 30 to 36 Gy in 1.8-Gy daily fractions, 5 fractions per week. Escalation treatments, including BEACOPP, and/or autologous stem cell therapies, were administered based on the response status at the end of ABVD therapy and decided by a multidisciplinary team. No therapy change was made on the basis of iPET unless progression was documented by CT.

Interim PET scans were performed 10–14 days after the second or fourth ABVD cycle while EoT PET scans were performed 3–8 weeks after the last ABVD cycle. The iPET findings were compared with EFS obtained based on routine follow-up clinical examination, CT and/or FDG-PET/CT scans. EFS was calculated from the start of ABVD until the date of progression/relapse based on CT or FDG-PET/CT, persistent disease in a post-therapy residual mass based on biopsy or abnormal end-of-therapy FDG-PET with other clinical evidence suggesting residual disease, death from any cause or last follow up.

### FDG-PET/CT imaging

PET images were acquired 1 h after intravenous injection of 310–450 MBq (3–5 mBq/kg) of FDG using a dedicated PET/CT scanner (Biograph mCT 64; Siemens, Erlangen, Germany). Patients were requested to fast for at least 6 h before the scan. All recorded serum blood sugar levels were below 200 mg/dl. PET images were acquired in a 3D mode position from the skull base to mid-thigh (FlowMotion technology; table speed 1 mm/second equal to 3 min/bed). Ordered subsets expectation maximization (OSEM) was used for PET image reconstruction. Low dose CT without intravenous contrast was used for attenuation correction and anatomical localization.

### Image analysis and response assessment

All FDG-PET/CT studies were reviewed by two board-certified Nuclear Medicine physicians in consensus (FA & ANK), In case of no consensus, a third reader (AA) impression was used for agreement. Readers were blinded to the clinical data and the results of other imaging studies.

Maximum intensity PET, Sagittal, axial and coronal PET images were reviewed using Syngo via (Siemens Medical Solution, Knoxville, TN) software. The response status on iPET and EoT PET was defined according to the Deauville criteria which are based on 5 Deauville scores (DS)^2^. Patients were classified as having a complete metabolic response (CMR) if the PET findings were assigned a DS of 1–3 and nCMR if the DS was 4 and 5. The final response assessment after the completion of ABVD therapy was based on EoT PET and, if feasible biopsy confirmation of residual FDG findings with a DS 4 and 5. EoT PET positive lesions were biopsied if Deauville score was 4 or 5 and the FDG uptake corresponded to a measurable lesion on CT scan portion of the PET/CT study. Only two patients with Deauville score 4 were found false positive and biopsy revealed inflammatory changes.

The difference in the prognostic value of i-PET-2 and i-PET-4 was examined based on the results of 69 patients with i-PET-2 scans and 176 patients with i-PET-4 scans.

### Statistical analysis

Descriptive analysis and data frequencies were estimated. Logistic regression analysis was used to investigate the association between the iPET response and EoT response. The survival rate was derived using Kaplan–Meier curves and compared by the log-rank test. Cox regression hazard model, univariate analyses, for testing the associations of variables with event-free survival (EFS) and overall survival (OS) were performed for the known risk factors in HL including, age, stage, and lymphocytes counts, albumin, leukocytes counts and bulky mass. Multivariable analyses were performed for the significant factors in univariate analysis. An alpha of < 0.05 was considered statistically significant. The statistics were performed using SAS version 9.4 (SAS Institute Inc., Cary, NC).

The event was defined as progression/relapse based on CT or FDG-PET/CT apparent as an increase in lesion size and/or new lesion(s), persistent disease in a post-therapy residual mass based on biopsy or abnormal end-of-therapy FDG-PET with other clinical evidence suggesting residual disease or death from any cause. EFS was calculated from the start of treatment to any of the above-events or last follow-up. OS was calculated from the starting date of therapy to death from any cause.

### Ethical standards

All procedures performed in studies involving human participants were in accordance with the ethical standards of the institutional and/or national research committee and with the 1964 Helsinki declaration and its later amendments or comparable ethical standards. The Institutional review board (IRB) has reviewed and approved this research project (IRB: 19 KHCC 37).

## Results

### Patients

The study cohort was composed of 111 (45.3%) female and 134 (54.7%) male patients. The median age was 29 years (18–83), with 81.6% of patients ≥ 45-year-old. One-third of the patients (77 patients) were stage IV and presented with extranodal involvement (bone marrow and/or viscera). A bone marrow biopsy was positive for 26 patients. Thirty-one patients (12.7%) had a bulky mass measuring ≥ 10 cm. All patients were treated with upfront ABVD (4–8 cycles), of them, 64% (157/245) received six cycles and 24.5% (60/245) received eight cycles. Patients with favorable early-stage disease were given 4 cycles, while patients with unfavorable early-stage disease were given 4–6 cycles of ABVD. Patients with advanced-stage received 6–8 cycles of ABVD. Escalation therapy was performed for 33/245 patients. Table 1 presents all patients characteristics in detail.

**Table 1:** Patients characteristics.

Variable	patients no. (%)
**Gender**	
Female	111 (54.7)
Male	134 (45.3)
**Age**	
< 45 y	200 (81.6)
≥ 45 y	45 (18.4)
**Extranodal Involvment (ENI)**	
No	168 (68.6)
Yes	77 (31.4)
**Stage**	
I	13 (5.3)
II	91 (37.1)
III	64 (26.1)
IV	77 (31.5)
**Bulky mass**	
No	214 (87.3)
Yes	31 (12.7)
**No. of ABVD cycles**	
4 cycles	28 (11.5)
6 cycles	157 (64)
8 cycles	60 (24.5)

### Associations between interim PET and the end-of-treatment response

**Figure 1:**
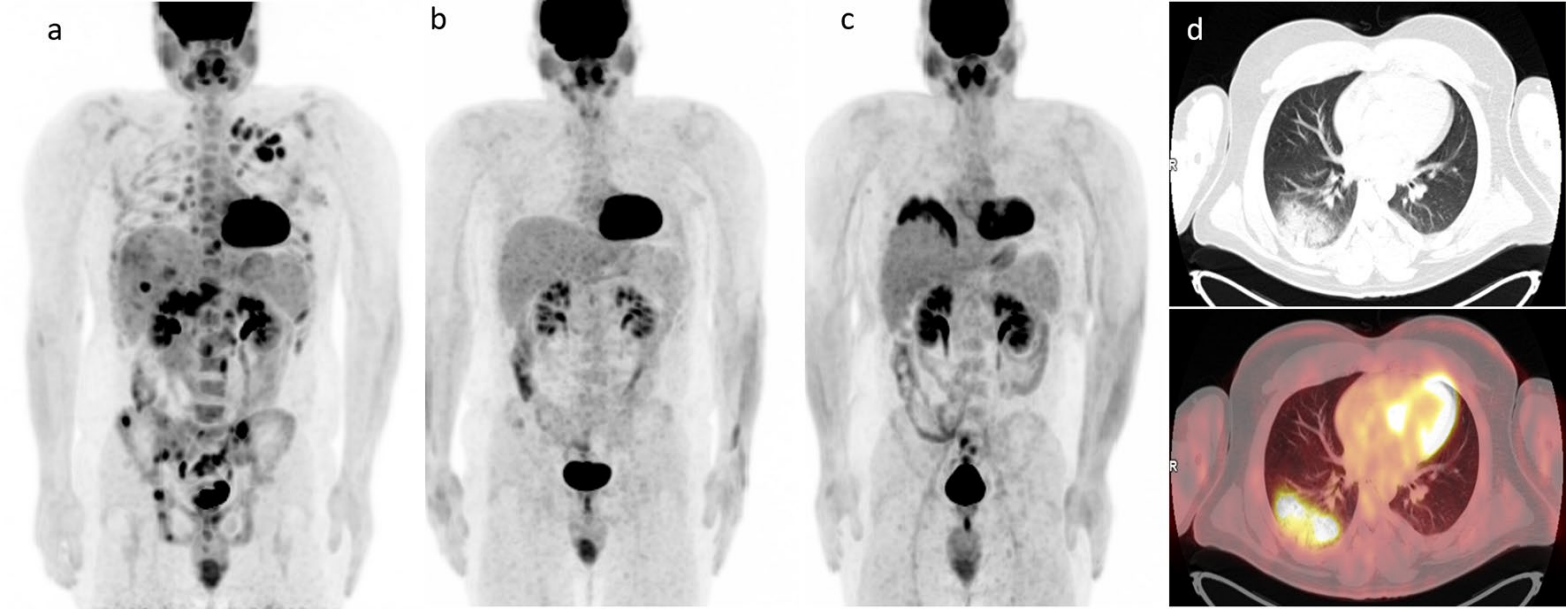
CMR on iPET & EoT PET. Baseline MIP FDG-PET (**a**) in a 26–year male patient, presented with extensive HL involving lymph node groups in both sides of the diaphragm with bone marrow involvement. MIP iPET after 2 cycles of ABVD (**b**) showing complete metabolic resolution of the hypermetabolic lymph nodes and the bone marrow lesion CMR (Deauville score 2). MIP FDG-PET after 4 additional cycles of ABVD (**c**) showed sustained CMR on EoT PET but with the development of inflammatory changes in the base of the right lung (**d**).

**Figure 2:**
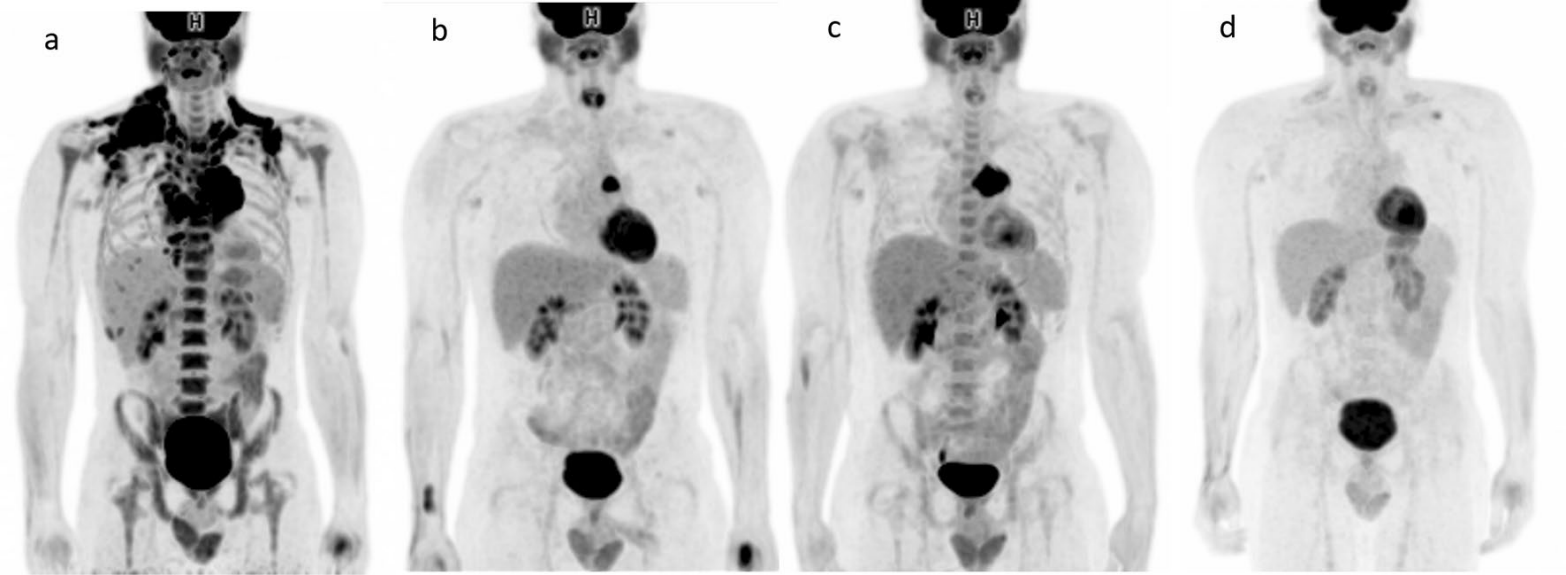
nCMR on iPET with further progression on EoT PET. Baseline MIP FDG-PET (**a**) in a 29-year male patient showing hypermetabolic lymphadenopathy involving multiple lymph node groups above the diaphragm (HL stage II). iPET after 4 cycles ABVD (**b**) showing nCMR with residual hypermetabolic enlarged lymph node in the left side of the mediastinum (Deauville score 5). Despite receiving 4 additional cycles of ABVD, the EoT PET (**c**) shows nCMR with further progression in the residual hypermetabolic mediastinal lymph node. Subsequently, the patient underwent a bone marrow transplant and CMR was achieved as demonstrated by the follow-up PET (**d**).

Among the 201 patients with CMR on iPET, 194 (96.5%) patients presented CMR, (Fig. 1), and 7 (3.5%) n-CMR at the EoT PET. Also, 23/44 (52.3%) patients with nCMR on iPET had an n-CMR (Fig. 2), while 21/44 (47.7%) patients had a CMR at the EoT PET (*P*-value < 0.0001). In addition, the number of administered cycles and the advanced disease stage are significantly associated with the response status at the end of treatment (Table 2).

**Table 2:** Associations of the clinical variables and iPET response with the EoT response status.

Variable	EoT-CR	EoT-nCR	*P*-value
**Gender**			0.817
Female	98	13	
Male	117	17	
**Age**			0.096
< 45 y	172	17	
≥ 45 y	43	2	
**Stage**			0.002
I-III	155	13	
IV	60	17	
**Bulky mass**			0.067
No	191	23	
Yes	24	7	
**No. of ABVD cycles**			0.02
4 cycles	27	1	
6 cycles	140	17	
8 cycles	48	12	
**Interim PET**			0.0001
iPET-CMR	194	7	
iPET-nCMR	21	23	

### Baseline risk factors and iPET associations with EFS

After a median follow-up of 32 months (range, 6–81 months), 48 patients presented disease progression.

In univariate analysis, the following factors were significantly associated with EFS: disease stage, lymphocytes and albumin (*P*-value = 0.002, *P*-value = 0.021 and *P*-value = 0.027, respectively) (Supplementary Table 1). Patients with disease stage 4, lymphocytes < 8% and/or albumin < 4 presented worse EFS than those with low disease stage, normal lymphocytes and albumin level (Supplementary Fig. 1). Also, iPET presented a statistically significant association with EFS; patients with iPET CMR presented a better EFS rate of 91% 3-y EFS than those with iPET nCMR (41% 3-y EFS, *P*-value < 0.0001) (Supplementary Fig. 1).

On multivariable analysis, disease stage and iPET were independent factors for prediction of EFS (*P*-value = 0.008, *P*-value = 0.001, respectively) (Table 3).

**Table 3:** Multivariable Cox regression of the association between the the demographic variables, baseline clinical factors and iPET with EFS.

Multivariable analysis of event-free survival			
Variable	HR	95% CI	*P* value
No. of cycles	0.94	0.71—1.24	0.656
Stage (4 vs 1–3)	2.43	1.26—4.67	0.008
Lymphocytes (< 8% vs ≥ 8%)	1.51	0.74—3.09	0.258
Albumin (≥ 4 vs < 4) g/dl	1.62	0.88—2.97	0.118
iPET (nCMR vs CMR)	6.62	3.65—12.02	0.001

### Baseline risk factors and iPET associations with OS

A total of 21 patients died at the time of study analysis.

Multiple factors showed statistical significant associations with patient OS on univariate analysis, including age of the patient, disease stage, albumin level (*P*-value = 0.0001, *P*-value = 0.014 and *P*-value = 0.005).Patients with old age, advanced disease stage and low albumin level presented low survival probability than those with younger age, low disease stage and high albumin level, (Supplementary Fig.  2). Regarding the iPET, it also presented a significant association with OS: patients with iPET showed a better OS rate with 95% 3-y OS compared to those with iPET nCMR (86% 3-y OS, *P*-value = 0.0039) (Supplementary Fig. 2, supplementary Table 2).

On multivariable analysis, age, albumine level and iPET were independent predictive factors (*P*-value = 0.001, *P*-value = 0.048 and *P*-value = 0.002, respectively) (Table 4).

**Table 4:** Multivariable Cox regression of the associations between the demographic variables, clinical factors and iPET with patient overall survival.Multivariable analysis for overall survival.

Variable	HR	95% CI	P value
iPET (nCMR vs CMR)	4.57	1.77—11.82	0.002
Stage (≥ 4 vs 1–3)	1.75	0.70—4.35	0.229
Age (≥ 45 vs < 45) years	7.32	2.77—19.35	0.001
Albumin (≥ 4 vs < 4) g/dl	2.64	1.01—6.94	0.048

### Comparison between the prognostic value of iPET-2 and i-PET-4

**Figure 3:**
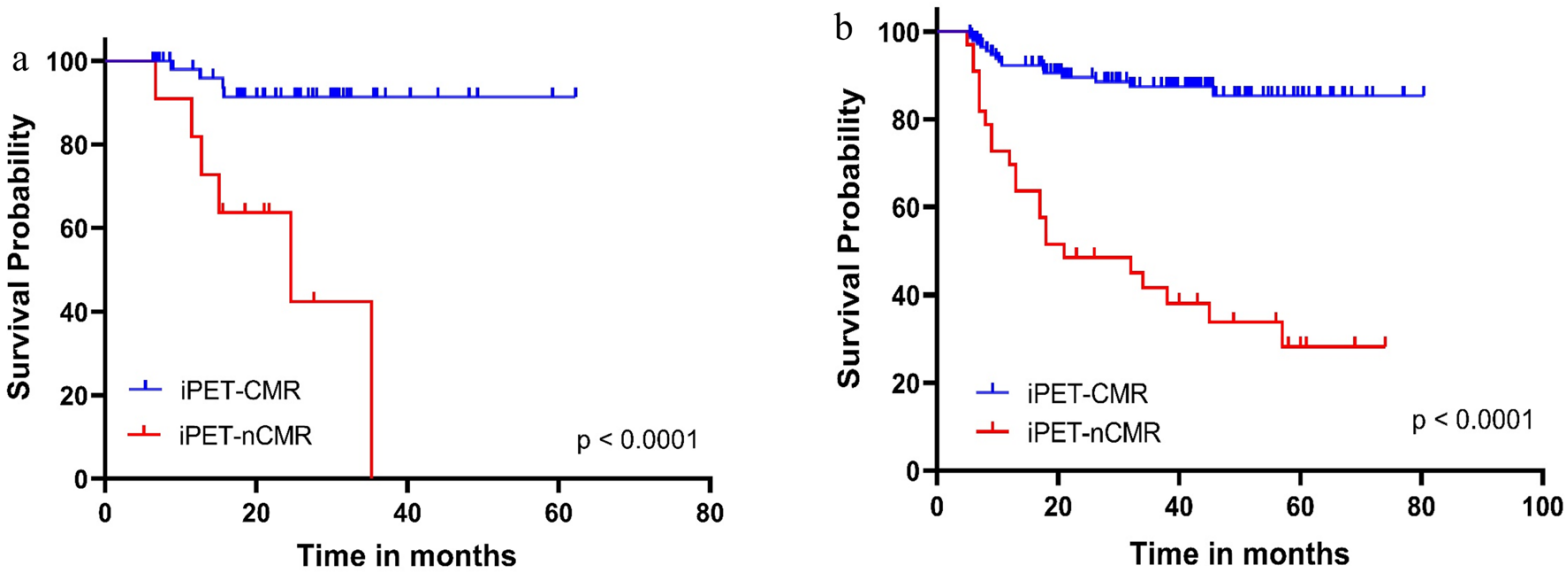
Kaplan Meier Plots of event-free survival in a subgroup of patients who underwent interim PET after 2 cycles (**a**) and 4 cycles (**b**). *P*-value derived from log-rank test.

Figure 3a shows the EFS of patients with CMR vs. nCMR in the 69 patients who underwent iPET-2. Figure 3b shows the EFS of patients with CMR vs. nCMR in the 176 patients who underwent iPET-4. No significant differences in the prognostic value are noted between iPET-2 and i-PET-4 with both showing highly significant differences in EFS between the CMR and nCMR patients (*P*-value < 0.0001): patients with iPET-2-CMR and iPET-2-nCMR had 3-y-EFS’s of 93.1% and 45.5%, respectively while the respective values for patients with iPET-4-CMR and iPET-4-nCMR were 89.5 and 39.4%.

## Discussion

The main objective of the current study was to assess the predictive value of iPET in HL treated with ABVD, the most widely used chemotherapy regimen for this disease.

Our study assessed the prognostic value of iPET in HL by requiring the use of the now-standard Deauville criteria for interim scan interpretation and that iPET is not employed to modify treatment based on the scan result which would potentially compromise the ability to assess its true predictive value. To our knowledge, this study is the first of its kind from the Arab region and one of only a few from non-Western developing counties^18,19^.

The most important finding of our investigation is that iPET performed after 2 or 4 cycles of ABVD strongly correlated with EoT response, EFS and OS. In multivariable analyses, only iPET (*P*-value = 0.001) and disease stage (*P*-value = 0.008) remained independent predictors for EFS whereas iPET (*P*-value = 0.002), age (*P*-value = 0.001) and albumin (P-value = 0.048) retained significance for OS. The independent prognostic value of iPET is consistent with previous reports where iPET was found to be a powerful predictor of outcome being superior to the well-established international prognostic score in advanced HL^7^. Similar findings were reported by Hutchings et al. who found iPET to be a strong independent predictor of progression-free survival in HL superior to both clinical stage and extranodal disease^6^.

The high negative predictive value (NPV) of iPET in HL of 91% using the 3 y-EFS endpoint in our study is consistent with what has been reported in the literature for both early- and advanced-stage HL^4–9,18–20^. The high percentage of negative iPET scans (82% in our study) combined with the high NPV of iPET lend support to trials aimed at ABVD treatment de-escalation in an effort to reduce toxicity while maintaining efficacy^10–15^.

The PPV of iPET in our study based on the 3-y-EFS was 59%, significantly lower than the NPV. In fact, 21 of 44 patients (48%) with an iPET-nCMR in our study converted into CMR at the end of therapy with all remaining in CR until their last follow-up. The lower PPV of iPET found in our study is, in general, consistent with the findings of prior studies, particularly in early-stage HL^4,5,16^. For example, a retrospective analysis of iPET-2 scans following 2 cycles of ABVD in an international cohort of 260 patients with advanced HL interpreted using the Deauville criteria yielded a PPV of 73% compared to a NPV of 94%^4^. Overall, these data suggest that treatment escalation may not always be appropriate based on the iPET scan result alone and that confirmation of residual disease by biopsy may sometimes be required prior to escalating treatment. This is reflected in the National Cancer Center Network (NCCN) clinical practice guidelines pertaining to treatment of HL where biopsy is either mandated or provided as an option for residual lesions with a Deauville score of 5 following 2 cycles of ABVD or other regimens^17^. This is because a positive biopsy in this setting would justify the administration of aggressive salvage chemotherapy that would otherwise be withheld if the biopsy is negative^21^.

A limitation of the current study is that the majority of interim PET scans were performed after 4 cycles of ABVD whereas the majority of more recent studies investigated PET after 2 cycles with some guidelines recommending iPET-2 as the optimal iPET scan^4–9,17–20^. In this respect, it is important to note that our study found no association between the timing of iPET and the iPET-response status. Moreover, we have not found significant differences in the prognostic value of iPET-2 and i-PET-4 with both showing highly significant differences in EFS between the CMR and nCMR patients. Previous studies in HL in which patients underwent both iPET-2 and i-PET-4 also did not find significant differences in the prognostic value between the two^6^.

Nevertheless, it is important to note that iPET is now recommended after 2 rather than 4 cycles of ABVD^17^ probably due to the fact that treatment modifications, if indicated, should take place as early as possible after the response assessment^6^.

It should also be emphasized that nowadays HL patients are given personalized treatment based on extensive data supporting the use of iPET to guide therapy and change management^10,11,13–17^. However, iPET-based personalized treatment has been recommended as standard of care only in recent years, for example in the NCCN clinical practice guidelines from 2020^17^. Our study includes patients treated between 12/2013 and 12/2017 before personalized treatment has been fully established as a new paradigm. Our data supports the use of personalized treatment in the Jordanian/Arab population and this treatment is now offered to all HL patients at our center.

One example of the use of i-PET for response-adapted treatment was the UK-led RATHL trial in 1214 patients with advanced-stage HL^10^. In this trial patients with negative i-PET (defined as D5PS scores of 1–3) after 2 cycles of ABVD were randomized to continue with 4 more cycles of treatment with the standard ABVD regimen or with Bleomycin (a drug known for pulmonary toxicity) omitted, for a total of 6 cycles. There was no significant difference in 3 year PFS between patients who received ABVD and those who received Doxorubicin, Vinblastine, and Dacarbazine (hazard ratio, 1.13; *P*-value = 0.35). Patients who received the 3-drug regimen had less infection, neutropenic fever, and pulmonary toxicity. Thus, a negative iPET-2 can be used to spare patients Bleomycin in cycles 3 through 6 with maintained treatment efficacy and reduced toxicity.

## Conclusion

In conclusion, this study investigated the prognostic value of iPET interpreted using the contemporary Deauville criteria in HL patients receiving ABVD treatment unequivocally demonstrating its prognostic value of both EFS and OS and prediction of the treatment response. The study lends support to clinical trials investigating the merits of ABVD de-escalation when iPET is negative and indicates that positive iPET is not always associated with adverse outcomes and may, in certain scenarios require confirmation by biopsy before treatment escalation is contemplated.

## Supplementary Information

Supplementary Information.

## Data Availability

The datasets during and/or analyzed during the current study are available from the corresponding author on reasonable request.
