# Supplementary material for: Interim FDG-PET/CT for therapy monitoring and prognostication in Hodgkin’s Lymphoma
Source: Sci Rep. 2022 Oct 21;12:17702. doi: 10.1038/s41598-022-22032-3 (PMC9587214; doi:10.1038/s41598-022-22032-3)
Supplement: Supplementary file 1 — Supplementary Information. [file 41598_2022_22032_MOESM1_ESM.pdf]

Supplementary Figure 1. Kaplan Meier plots for the event-free survival according to the factors that presented significant association on univariate analysis: interim PET (a), disease stage (b), albumin level (c) and lymphocyte percentage of white cell count (d). P-values were calculated from the log-rank test.

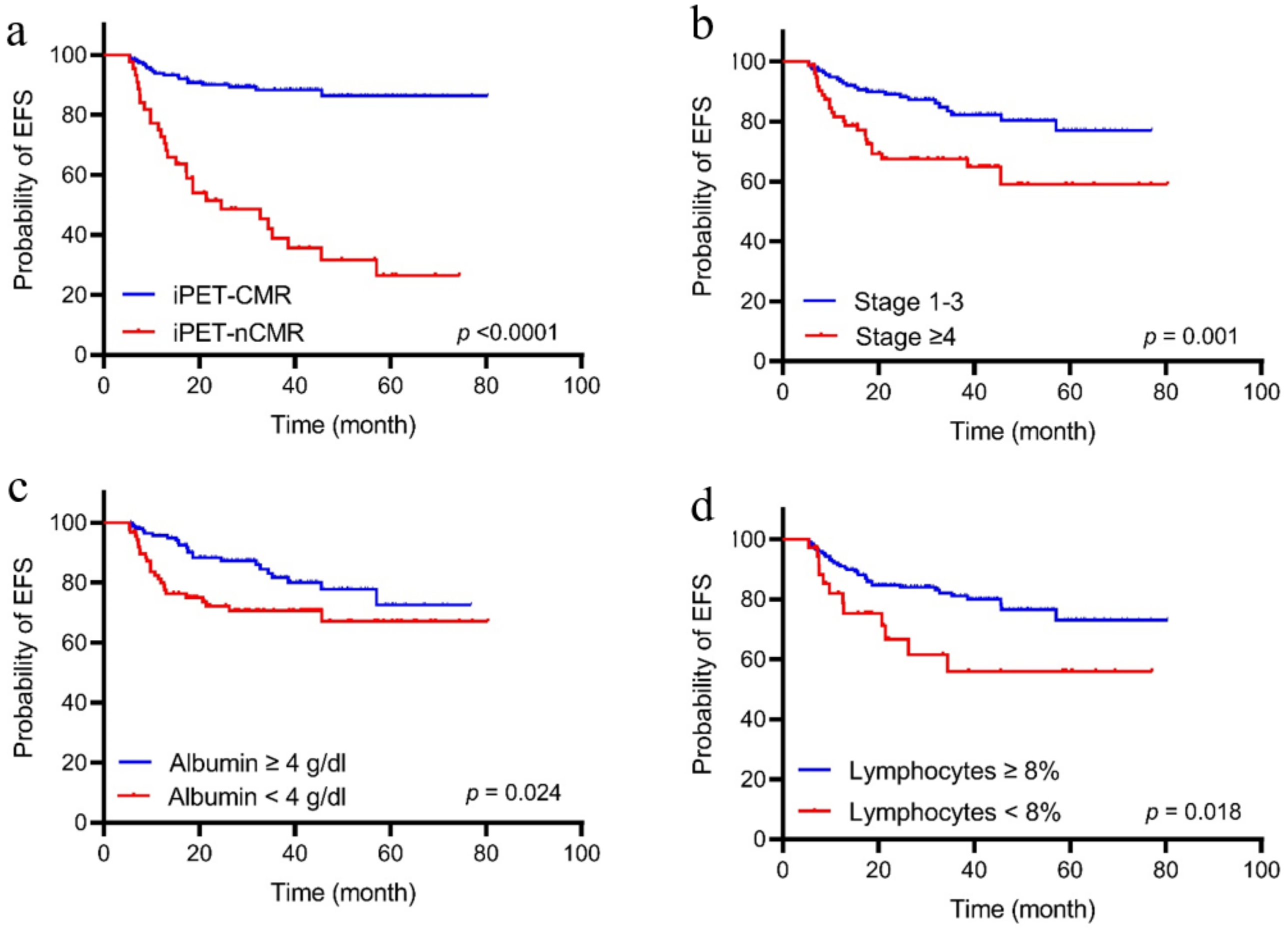

Supplementary Figure 2. Kaplan Meier plots for the overall survival according to the factors that presented significant association on univariate analysis: interim PET (a), disease stage (b), age (c) and albumin (d). P-values were calculated from the log-rank test.

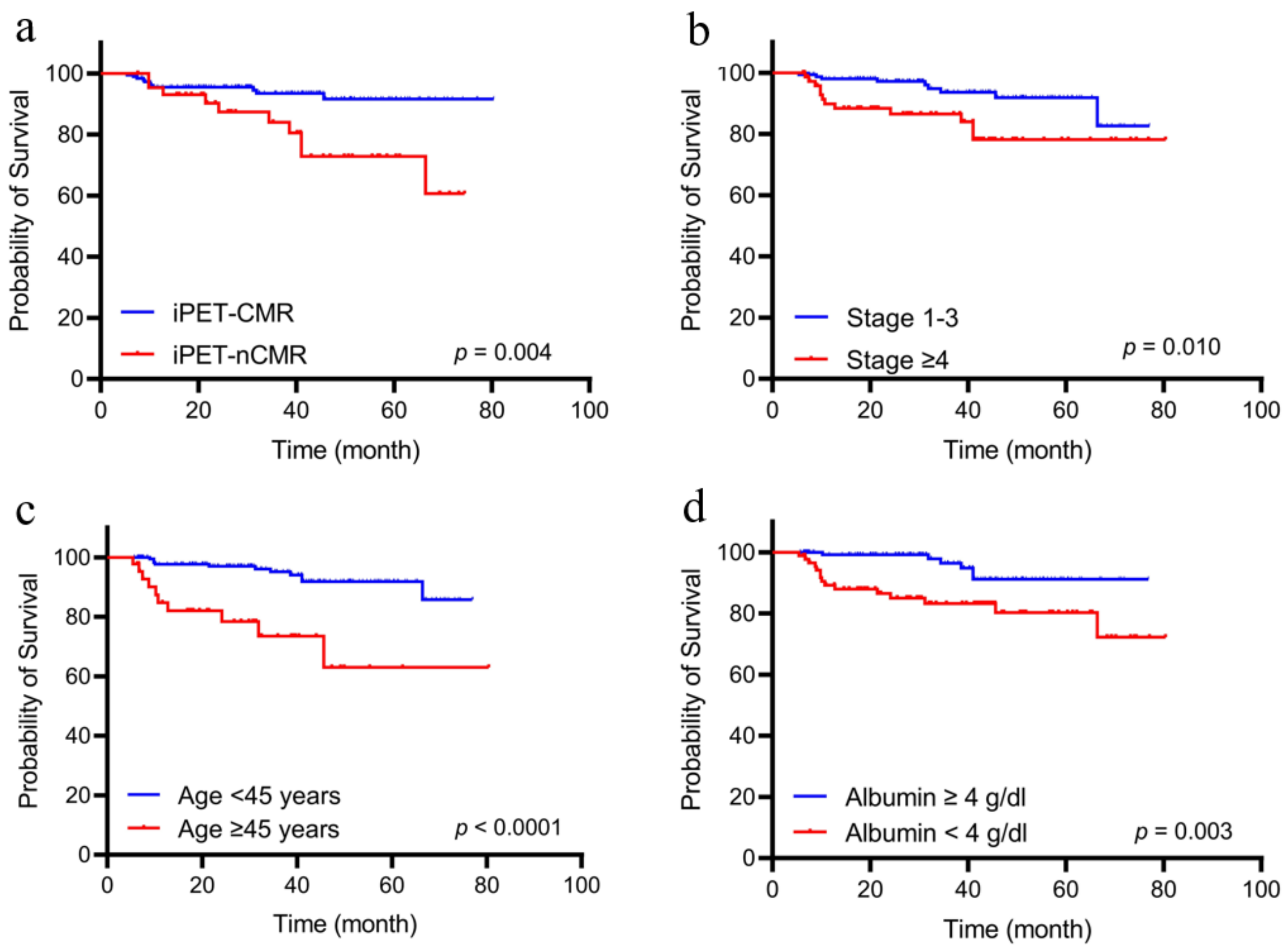

Supplementary Table 1. Univariate Cox regression analyses of the association of the demographic variables, clinical factors and iPET with the event-free survival.

Univariate analysis of event-free survival			
Variable	HR	95% CI	P-value
iPET (nCMR vs CMR)	7.08	3.98 - 12.58	0.0001
Stage (4 vs 1-3)	2.45	1.39 - 4.33	0.002
Lymphocytes (<8% vs ≥8%)	2.16	1.12 - 4.17	0.021
Albumin (≥4 vs <4) g/dl	1.90	1.08 - 3.35	0.027
No. of cycles	1.36	1.07 - 1.75	0.014
Leukocytes (≥15x10^3 vs <15x$10^{3)}$	1.52	0.85 - 2.71	0.156
Hemoglobin (<10.5 vs ≥ 10.5) g/dl	1.74	0.97 - 3.12	0.064
Age (≥45 vs <45) years	1.39	0.69 - 2.79	0.356
Gender (male vs female)	1.02	0.58 - 1.81	0.935
Bulky (yes vs no)	1.94	0.94 - 4.00	0.075

Supplementary Table 2. Univariate Cox regression analysis of the associations between demographic variables, clinical variables and iPET and patient overall survival.

Univariate analysis for overall survival			
Variable	HR	95% CI	Pvalue
Albumin (≥4 vs <4) g/dl	3.87	1.50 - 10.01	0.005
Hemoglobin (<10.5 vs ≥ 10.5) g/dl	1.59	0.66 - 3.85	0.302
Leukocytes (≥15x10^3 vs <15x10$^{3)}$	0.82	0.32 - 2.11	0.675
Lymphocytes (<8% vs ≥8%)	2.43	0.94 - 6.30	0.067
Gender (male vs female)	2.18	0.84 - 5.62	0.107
Age (≥45 vs <45) years	5.72	2.40 - 13.59	0.0001
Stage (4 vs 1-3)	2.96	1.24 - 7.04	0.014
Bulky (yes vs no)	0.32	0.04 - 2.43	0.273
iPET (nCMR vs CMR)	3.30	1.40 - 7.81	0.006
